# Meeting Report: 1st International Functional Metagenomics Workshop May 7–8, 2012, St. Jacobs, Ontario, Canada.

**DOI:** 10.4056/sigs.3406845

**Published:** 2013-04-15

**Authors:** Katja Engel, Deborah Ashby, Sean F. Brady, Don A. Cowan, John Doemer, Elizabeth A. Edwards, Klaus Fiebig, Eric C. Martens, Dennis McCormac, David A. Mead, Kentaro Miyazaki, Gabriel Moreno-Hagelsieb, Fergal O’Gara, Alexandra Reid, David R. Rose, Pascal Simonet, Sara Sjöling, Kornelia Smalla, Wolfgang R. Streit, Jennifer Tedman-Jones, Svein Valla, Elizabeth M. H. Wellington, Cheng-Cang Wu, Mark R. Liles, Josh D. Neufeld, Angela Sessitsch, Trevor C. Charles

**Affiliations:** 1 University of Waterloo, Waterloo, ON, Canada; 2 Health Canada, Ottawa, ON, Canada; 3 Rockefeller University, New York, NY, USA; 4 University of Pretoria, Pretoria, South Africa; 5 Waterloo Institute for Sustainable Energy, Waterloo, ON, Canada; 6 University of Toronto, Toronto, ON, Canada; 7 Ontario Genomics Institute, Toronto, ON, Canada; 8 University of Michigan Medical School, Ann Arbor, MI, USA; 9 Lucigen Corporation, Middleton, WI, USA; 10 National Institute of Advanced Industrial Science and Technology (AIST-Hokkaido), Sapporo, Hokkaido, Japan; 11 Wilfrid Laurier University, Waterloo, ON, Canada; 12 National University of Ireland, Cork, Cork, Ireland; 13 Natural Sciences and Engineering Research Council of Canada (NSERC), Ontario Regional Office, Mississauga, ON, Canada; 14 University of Lyon, Lyon, France; 15 Södertörn University, Huddinge, Sweden; 16 Julius Kühn-Institute, Braunschweig, Germany; 17 University of Hamburg, Hamburg, Germany; 18 MITACS, Waterloo, ON, Canada; 19 Norwegian University of Science and Technology, Trondheim, Norway; 20 University of Warwick, Coventry, UK; 21 Auburn University, Auburn, AL, USA; 22 AIT Austrian Institute of Technology GmbH, Tulln, Austria

**Keywords:** International functional metagenomics, metagenomics community, metagenomic libraries, sharing

## Abstract

This report summarizes the events of the 1^st^ International Functional Metagenomics Workshop. The workshop was held on May 7 and 8, 2012, in St. Jacobs, Ontario, Canada and was focused on building an international functional metagenomics community, exploring strategic research areas, and identifying opportunities for future collaboration and funding. The workshop was initiated by researchers at the University of Waterloo with support from the Ontario Genomics Institute (OGI), Natural Sciences and Engineering Research Council of Canada (NSERC) and the University of Waterloo.

## Introduction

An enthusiastic group of individuals with interests in functional metagenomics research convened in St. Jacobs, Ontario, Canada, for two days of intensive discussions on metagenomics. The participants included 26 attendees from academia, funding agencies and industry. The 1^st^ International Functional Metagenomics Workshop (IFMW) was organized and hosted by researchers from the University of Waterloo and funded by the University of Waterloo, Ontario Genomics Institute (OGI), and Natural Sciences and Engineering Research Council of Canada (NSERC). Its purpose was to identify challenges and opportunities and determine the best ways for the community to work together to achieve its goals. An important aspect of these discussions was the implementation of mechanisms for sharing resources such as metagenomic libraries, and the establishment of standards for library construction and data collection. A number of natural relationships have already formed among practitioners of functional metagenomics. By bringing people together, those relationships should be strengthened, resulting in improved collaborative opportunities. An important consideration to address at the outset was cooperation between industry and academia, and how to best structure activities of mutual benefit.

The 1^st^ IFMW had an international focus, and a central theme of this workshop was to identify solutions for a principle limitation of conventional functional metagenomics. Metagenomic libraries, which are difficult to construct, are typically project-specific and maintained in isolation. Each group has its own collection of libraries, and sharing between different research groups is limited. Importantly, a functional metagenomic library sharing model, “open resource metagenomics”, was presented for adoption by the community [1]. The overall focus of the workshop on functional metagenomics was distinguished from the broader metagenomics discipline, which more commonly centers on next-generation sequencing and bioinformatics, and is supported by networks such as Terragenome and the Earth Microbiome Project.

The talks on both workshop days were divided into thematic sessions. Workshop speakers covered important issues within the scope of each session topic and generated ideas for the open discussions that followed. Several workshop attendees were involved in moderating open group discussions on both days so participation by attendees was high.

## Day 1

### Session I. Introduction to functional metagenomics

Trevor Charles (University of Waterloo, Waterloo, ON, Canada) opened the workshop by welcoming participants and presenting an overview of the planned sessions and goals: bringing people together, strengthening relationships, identifying challenges and solutions, discussing implementation of a system to share libraries and set up collaborative opportunities. Eight topical sessions were designed around the same basic questions for each: What are the major challenges? How are these challenges being addressed? What improvements are necessary? Can we collaborate to do this better?

The next speaker of the day was Klaus Fiebig (Ontario Genomics Institute; OGI; Toronto, ON, Canada), who described Genome Canada funding opportunities related to metagenomics. Klaus emphasized that government funding is strongly focused on applied research. He outlined how Genome Canada funding opportunities work as “matching” programs: the government provides half of the funds, matched by another partner that provides either cash or in-kind contributions. Fergal O’Gara (University of Cork, Cork, Ireland) added that discussions regarding basic versus applied research are ongoing in Europe as well. He was critical of the applied nature of funding for metagenomics, stating that this is still a young field and much basic groundwork is still required; metagenomics may not have yet approached the point of applied research. His sentiments were echoed by several of the other workshop attendees. Alexandra Reid (Natural Sciences and Engineering Research Council of Canada; NSERC), followed Klaus’ talk with an overview of funding opportunities at NSERC that also have requirements for commercial partnerships and matching funds.

Trevor Charles presented an historical overview of functional metagenomics and then considered the question of how to bring this research community together. He introduced the Canadian MetaMicroBiome Library (CM^2^BL) initiative [2] as an example of open-resource metagenomics that was motivated to unite the research community around shared tools and resources.

Gabriel Moreno-Hagelsieb (Wilfrid Laurier University, Waterloo, ON, Canada) focused his talk on the major issues related to metagenomic annotation, data sharing, and curation. He pointed out that low sequencing costs have resulted in vast amounts of DNA sequence data and many draft genomes to which metagenomic sequence data can be compared, but few serious efforts to bring these genomes to *finished* quality, and this affects the quality of sequence databases that contribute to sequence-based metagenomics analysis. He emphasized the need for computer scientists to contribute to the field of metagenomics, and the need for biologists to communicate effectively with these experts.

The discussion at the end of the first session was chaired by Trevor Charles and focused on the need for computer scientists within our field and raised questions about how to recruit and integrate them. Furthermore, the discussion suggested a possible need for a reward system for curation and annotation to help attract participation in this field of work by young computer scientists.

### Session II. Metagenomics technology overview

The second session of Day 1 began with a talk by Sean Brady (Rockefeller University, New York, NY, USA), who presented details of library construction and small molecule screening. He demonstrated that systematic library screenings enabled him to find new gene clusters and novel compounds with new or rare molecular arrangements.

The next presenter was Don Cowan (University of Pretoria, Pretoria, South Africa) who talked about functional enzyme screening and shared his experiences, including examples of successes and failures. His work on high throughput expression screening showed that the efficiency of finding positive clones varied enormously between different enzyme classes due to the lack of inducers or co-inducers, failure of some enzymes to fold correctly, and toxicity of some gene products to the host.

Kentaro Miyazaki (National Institute of Advanced Industrial Science and Technology; AIST-Hokkaido; Sapporo, Hokkaido, Japan) presented his work on host engineering of *Escherichia coli* to solve the expression problems of metagenomic libraries, most notably his ability to engineer ribosomes to improve *E. coli* as an expression host.

The discussion following the session was chaired by Sean Brady. One question raised was which user-friendly bioinformatics tools are available and easy to use for novices? How long will it be until a universal expression platform is developed? How much can Kentaro Miyazaki’s research contribute in terms of generating a universal system? It was generally agreed that, at present, a combination of different hosts may be the best solution. Sean Brady suggested that a long-term goal would be to be able to test “a hundred different pathways in a hundred different bacteria”.

### Session III. Applied aspects of functional metagenomics

Elizabeth Edwards (University of Toronto, Toronto, ON, Canada) started the session with an example of applied metagenomics in bioremediation of chlorinated ethene contaminated sites. This work was an example of a successful university-industry partnership but required over 10 years to yield commercial value. This is not consistent with expectations of funding agencies, which often expect commercialization arising from partnerships within a few years. Are there strategies that could reduce this time frame?

Wolfgang Streit (University of Hamburg, Hamburg, Germany) spoke about challenges in functional and applied metagenomics and his experiences with industry partnerships. He emphasized that it takes up to 3 or 4 years to identify novel enzymes, to develop screens, to characterize and then provide enzymes on a large scale. He was able to give a few examples of successful projects from his long experience with industry-academia relationships.

Josh Neufeld (University of Waterloo, Waterloo, ON, Canada) talked about the coupling of stable-isotope probing (SIP) and functional metagenomics. He illustrated how culturing captures a few microorganisms, drawing selectively from both abundant and low-abundance organisms; next-generation sequencing of bulk DNA captures data from predominant organisms; methods such as cell sorting and SIP can capture both abundant and rare microorganisms, but offers a less biased and more targeted approach. He gave an example of recent work involving multiple soils and isotope-labeled substrates as a collaboration with an industrial partner (Iogen).

The session was concluded with a discussion chaired by Elizabeth Edwards. Questions debated included: Do more functionally distinct results emerge from SIP than from enrichment culture? What is the rare biosphere? Which high-throughput methods are available and what are the bottlenecks? And again, the need for new expression platforms was vocalized as an important issue in metagenomics. Wolfgang Streit mentioned that there is a collaborative arrangement in Germany where participating labs develop 2-3 host backgrounds for protein expression. However, he is not aware of any international collaboration, but it is evident that there is much interest in such efforts.

### Session IV. Metagenomics and major questions in microbial ecology I

Session IV was started with Pascal Simonet (University of Lyon, Lyon, France) who introduced the Terragenome project, an international consortium that seeks the complete sequencing of reference soil metagenomes. The first reference site was Rothamsted (UK) but funding problems became evident due to work with an out-of-country soil sampling site.

Sara Sjöling (Södertörn University, Huddinge, Sweden) spoke about soil and sediment functional metagenomics. She introduced the MetaExplore consortium that screens for enzymes of industrial interest as well as the Baltic Sea metagenomics project that seeks to answer ecological questions through sequence analysis and functional metagenomics.

Svein Valla (Norwegian University of Science and Technology, Trondheim, Norway) then reported on issues that occurred when constructing a new vector that was able to harbor up to 200 kb inserts. This vector would occasionally show an insertion of *E. coli* genomic DNA introduced during host transfers. He also talked about the growing fish farming industry in Norway, a project to look for microbial producers of astaxanthin in metagenomic libraries and reports about issues with reproducibility of metagenomic screens.

Fergal O’Gara (National University of Ireland, Cork, Ireland) spoke about rhizosphere and marine metagenomics. He highlighted that just as genomics technologies contributed to our understanding of the bacterial genetic determinants of metabolic processes within the context of cultured microorganisms, comparative metagenomics should be leveraged to do the same; to identify important determinants of community metabolism. He also introduced the European Union collaborative project MaCuMBA with 23 participants who work on improving culture media, co-cultivation approaches and high-throughput isolation methods.

The facilitated discussion was chaired by Pascal Simonet. Discussions involved topics such as movement of libraries into different hosts, storage of libraries, construction of more difficult BACs, and possible uses of the Phylochip or functional gene arrays.

## Day 2

The morning included the second part of the “metagenomics and major questions in microbial ecology” sessions, followed by a session on open resource metagenomics. The second half of the day concentrated on metagenomics and industry as well as funding opportunities and strategies.

### Session V. Metagenomics and major questions in microbial ecology II

The session was opened by Elizabeth Wellington (University of Warwick, Coventry, UK) who highlighted the use of metagenomics for bioexploration, the search for novel enzymes and new resistance genes. She explained the effort to identify family 19 chitinases from soil using metagenomic and metaproteomic approaches.

Eric Martens (University of Michigan Medical School, Ann Arbor, MI, USA) gave a presentation about carbohydrate metabolism in the human microbiome. He emphasized that members of the *Bacteroidetes* can be genetically manipulated and would be promising hosts for functional metagenomics.

Kornelia Smalla (Julius Kühn-Institut, Braunschweig, Germany) spoke about direct capturing of plasmids in different recipient strains as a cultivation-independent means to obtain genes and complete operons, localized on mobile genetic elements, directly from environmental bacteria. The application of direct capture methods furthermore assists in identifying new plasmids, adapted to different hosts. Thus environmental mobilome recovery strategy is considered to be complementary to functional metagenomics.

The session was concluded with a discussion chaired by Elizabeth Wellington. She pointed out significant challenges in metagenomics such as: What are the best methods for capturing the mobilome? How does one capture phages? Which methods exist to rescue genomic islands? Which are the best methods to obtain high quality high molecular weight DNA? How does one capture inactive bacteria from soil? Are there new methods to introduce DNA into cells? One question revisited from earlier: Have there been efforts to identify the best microorganisms for expression screening? Can systematic experiments be done to determine this? Which traits are necessary? It was recommended to start an initiative where everyone can contribute their experimental results about hosts and traits.

### Session VI. Open resource metagenomics

Trevor Charles started the session with an introduction to the concept of “open resource” and its relationship to metagenomics. He talked about a model for sharing metagenomic libraries and proposed the use of large-insert libraries in versatile vectors, pooling clones for storage and distribution, along with extensive metadata and sequence-based characterization. Different models for library distribution were discussed as well as the Nagoya Protocol on Access and Benefit-sharing (October 2010, Nagoya, Japan), which could have a large impact on metagenomics, once ratified.

Josh Neufeld facilitated the discussion about open resource metagenomics and materials sharing agreements. With respect to the Nagoya protocol, Don Cowan pointed out that free sharing of materials is becoming highly restricted in law and very problematic in many countries such as South Africa, Zambia and New Zealand. On the other hand, free movement of genetic materials in Europe was seen as more permissible. Metagenomes should be exempt from those restrictions but this is not likely to be granted by governments. This could develop into a huge impediment for international functional metagenomics efforts.

### Session VII. Metagenomics and industry

David Mead (Lucigen Corporation, Middleton, WI, USA) started the metagenomics and industry session. He provided information about his corporation as well as its key interests and projects. He summarized new Lucigen tools available for metagenomics. He encouraged the community to share knowledge so that the same screening mistakes are not repeated.

Trevor Charles talked about his collaboration with Iogen Corporation for screens of metagenomic libraries for industrially relevant enzymes. This type of collaboration is necessary for many of the current funding models that require matching funds or in-kind contributions. Recent changes to Iogen’s energy-related enzyme operations involved large company lay-offs. This impacted the collaboration with the University of Waterloo as the Iogen collaborators left the company. This example illustrates some of the perils of linking the majority of research funding to industry.

The discussion was chaired by Eric Martens and one of the first questions debated was how to set up industry-academia relationships in the first place. It was mentioned that many US universities set up restrictions for collaborations with industry. Canadian institutions set their own rules and at the University of Waterloo researchers own what they discover, although it also depends on the company partner. John Doemer (Waterloo Institute for Sustainable Energy; WISE; Waterloo, ON, Canada) described his work at WISE that focuses on making initial contacts between industry and academia as well as help with maintaining relationships, IP issues, and funding resources. Because most Canadian research funding is tied to academia-industry partnerships, there is high demand for centers that create contacts between those partners.

### Session VIII. Funding opportunities and strategies

Angela Sessitsch (AIT Austrian Institute of Technology GmbH, Tulln, Austria) spoke about the importance of collaborations, showing how research is more successful when combining complementary expertise, sharing tools and avoiding repeated mistakes. Funding is an issue for international collaborations and a task force group among EU/US/Canada is recommended to explore funding opportunities.

Mark Liles (Auburn University, Auburn, AL, USA) presented on academic-industry partnerships and funding opportunities. A quick survey within the group of participants showed that all researchers (23) had government funding, 6 had funding from industry, 5 had university funding, and 1 had private funding. The question of how to integrate the academic work with industry was again raised because academia and industry have fundamentally different cultures. Industry values revenue and application of research but academia values discoveries (i.e. publications), often without regard to application or potential commercialization.

Mark Liles chaired a discussion and Elizabeth Wellington emphasized that the time spent securing funds from a company can negatively impact an academic career. This takes time away from writing papers or grants for traditional funding sources, which affects the scientist's incentive to form industry relationships. Nevertheless, partnerships are often advantageous because scientists with industry support and partnerships can publish at higher rates, patent more frequently, have higher income and participate in more professional activities. In addition, industry gains access to resources, expertise, IP, skilled personnel, and enhanced competency leading to new business opportunities.

## Meeting closure and next steps

During the workshop and discussions it became apparent that there are a number of shared interests within the functional metagenomics community, and there is considerable potential value in strengthening those community interactions. There was clearly a high level of interest in establishing a metagenomics consortium with annual meetings in member countries. A geographically representative task force for the establishment of the consortium was formed by a group nomination process. The task force consists of the following representatives from research and industry: Don Cowan, Mark Liles, David Mead, Angela Sessitsch, Kentaro Miyazaki, and Trevor Charles. The 1^st^ International Functional Metagenomics Workshop was concluded by Trevor Charles. He thanked everyone for their active participation and looked forward to future consortium activities.
